# Supplementary material for: Synthesis and Characterization of a Self-Polycondensation Diazaphthalanone Monomer and Its Polymers from Polycondensation Reactions
Source: Polymers (Basel). 2022 Sep 19;14(18):3904. doi: 10.3390/polym14183904 (PMC9502096; doi:10.3390/polym14183904)
Supplement: Supplementary file 1 [file polymers-14-03904-s001.zip › polymers-1898332-supplementary.pdf]

Supporting Information

Synthesis and Characterization of a self-Polycondensation diazaphthalanone monomer and Its Polymers from Polycondensation Reactions

Xin Liu [1], Xiaozhou Zhang [1,2,*], Jiawei Jiang [1], Hongge Jia [1,2], Xigao Jian [3] and Jinyan Wang [3]

[1] College of Materials Science and Engineering, Qiqihar University, Wenhua Street 42, Qiqihar 161006, China
[2] Heilongjiang Province Key Laboratory of Polymeric Composition Material, Qiqihar 161006, China
[3] Department of Polymer Science & Engineering, Dalian University of Technology, Dalian 116024, China
* Correspondence: zhangxzh-n@163.com

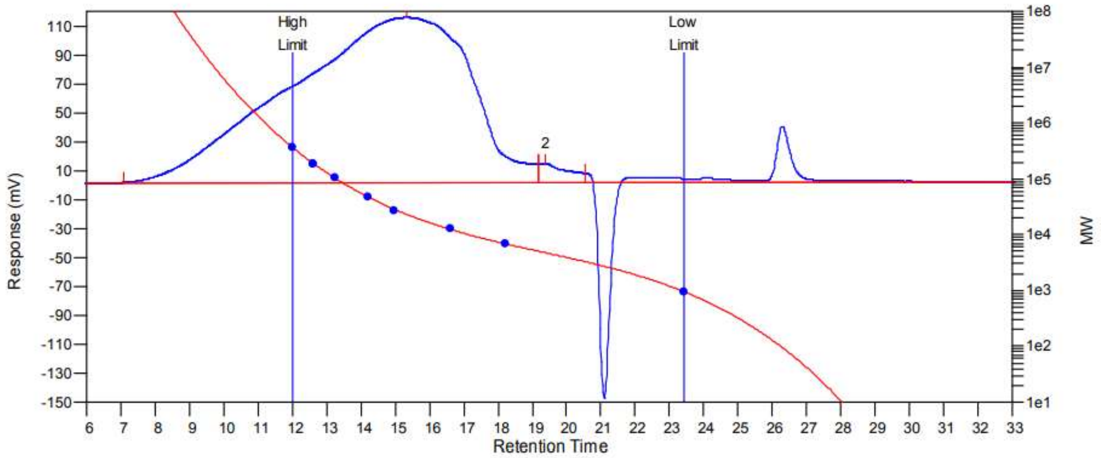

Figure S1. GPC of PCDD

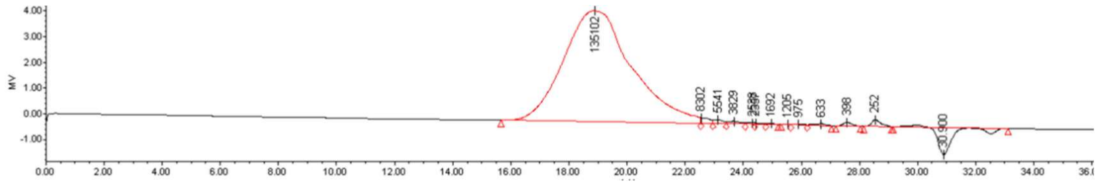

Figure S2. GPC of PBCD

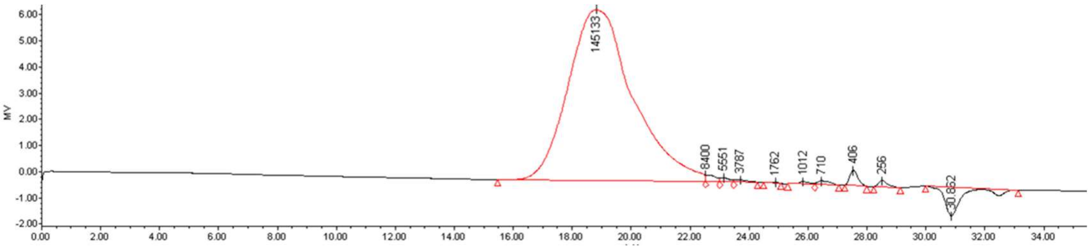

Figure S3. GPC spectra of PBD
